# Enablers to high vaccination uptake among a disadvantaged minority population: a qualitative study of the Arab population of Israel

**DOI:** 10.1186/s13584-024-00660-6

**Published:** 2024-12-05

**Authors:** Jumanah Essa-Hadad, Danielle E.M.C. Jansen, Johanna P.M. Vervoort, Michael Edelstein

**Affiliations:** 1 Azrieli Faculty of Medicine, Bar Ilan University, Safed, Israel; 2 Department of General Practice and Elderly Care Medicine, University Medical Center Groningen, University of Groningen, Groningen, 9712 CP The Netherlands; 3 Department of Health Sciences, University Medical Center Groningen, University of Groningen, Groningen, 9712 CP The Netherlands

**Keywords:** Vaccination, MMR, HPV, Children, Healthcare system enablers, Arab minority, Israel

## Abstract

**Background:**

Over 1.9 million Arabs live in Israel and constitute 21% of the total population. Despite being a disadvantaged minority population with wide gaps in health indicators, Arabs have higher Human Papillomavirus (HPV) and Measles, Mumps, and Rubella (MMR) vaccination rates compared with the general Jewish population.

**Methods:**

In-depth interviews with 21 health care providers, 16 Arab mothers, and 16 teenagers were conducted to collect information about health system enablers to HPV and MMR vaccination. All interviews were conducted in Arabic by an Arab researcher, audio-recorded, transcribed, and analysed using thematic analysis of the transcripts. Themes were mapped according to the WHO Health Systems Building Block Framework.

**Results:**

We identified several health system enablers. On the service delivery level, accessible and availability of vaccination services, delivery of vaccines through the school system and mother child clinics, and framing vaccinations as the norm were themes. Personable characteristics of the health workforce, the health care providers belonging to the same cultural group, and high levels of trust towards healthcare professionals were reported. Vaccination provided at no cost was also identified. On the leadership/governance level, the Arab community felt equal treatment and distribution of services, which was also an enabler reported. Despite high vaccine uptakes, parents and teenagers had limited knowledge regarding vaccination, particularly HPV.

**Conclusions:**

This study highlights that a combination of good access and delivery to vaccination, delivered by a culturally competent, available, accessible respectful workforce can enable disadvantaged minorities to achieve high vaccine coverage, in particular in a cultural context where the population trusts and follows medical advice. Such evidence can serve as a basis for developing policies, interventions, and guidance to improve vaccine uptake among other underserved minority communities.

## Introduction

Achieving and sustaining high vaccine uptake in the European region requires addressing the specific challenges that underserved populations face with regards to immunization. The World Health Organization (WHO) Immunization Agenda 2030, the global immunization strategy for the current decade, has for the first time explicitly placed equity as a strategic priority for immunization programmes [1]. Groups that are underserved such as socioeconomically disadvantaged populations, cultural or religious minorities, and migrants, achieve substantially lower vaccine uptake compared to the general population [2]. For example, in the Netherlands in 2017, HPV vaccine uptake among Turkish and Moroccan adolescents was 23% and 31%, respectively, lower than in other adolescents [3]; in Greece in 2019, uptake for measles was 55% lower in migrant children aged 0–4 than in other children of the same age [4]. Barriers to vaccination among underserved populations include language issues, access limitations, literacy limitations, financial and administrative problems, insufficient vaccination regulations, lack of knowledge about health, and lack of health care professionals trained to provide culturally sensitive care [5–7].

Some socio-economically disadvantaged populations manage however to achieve uptake similar or higher than the general population [8, 9]. An emerging approach that has been used to describe health inequity and provide recommendations for improvement is positive deviance, a bottom–up approach that identifies and learns from those who demonstrate exceptional performance on an outcome of interest [10]. The approach assumes that problems can be overcome using solutions that already exist within communities. Despite facing the same constraints as others, ‘positive deviants’ identify these solutions and succeed by demonstrating uncommon or different behaviours [11]. The Arab minority in Israel is one such example owing to their disadvantaged status and high vaccination coverage. Arabs constitute 21% of the Israeli population (1.9 million citizens) and are split between a Muslim majority and a Christian minority. Most Arabs live in Israel’s social and geographic periphery, which impacts their socio-economic status [12–14]: 35.8% live below the poverty line compared to 17.7% of the Jewish population [15]. Only 53% of Arab adults, compared to 83% of Jewish adults, participate in the labor force, mainly due to low participation of women. Only 36% of Arab adult women work, compared to 82% of Jewish women. Household income is little over half of that of the Jewish population [13]. Despite universal coverage and national health insurance law for all citizens since 1995, health inequities between Jews and Arabs persist [16]. For example, life expectancy among Arab men and women was 78.1 and 81.7 years, respectively, compared to 81.7 and 85.1 years in the Jewish population. Infant mortality rate among Arabs was 5.2 per 1,000 births, compared to 2.3 among Jews.

Despite low socio-economic status and poorer health outcomes on many levels, evidence from official reports and research shows that Arab children achieve higher coverage for routine vaccines in comparison to the general Jewish population. A 2018 Israel Ministry of Health report reported that 95% of Arab children were fully vaccinated by age 17, compared to 91% of Jewish children [17, 18]. A 2019 study showed that in Jerusalem MMR vaccine coverage was higher Arab children than Jewish children at any age [17, 18]. In 2018, coverage of MMR vaccine by 13 months was 82% among Arabs compared to 61% among Jews [19]. Consent rate to the HPV vaccine was 98.8% among Arabs compared to 54.3% among Jews [20].

While research has focused on barriers to vaccination among underserved populations, there are a few studies that report health system vaccination enablers through the prism of positive deviance. We identified and described healthcare system enablers to child vaccination, specifically for MMR and HPV vaccines in the Arab population in Israel. We chose these two vaccines to cover different ages at which individuals receive vaccines according to the lifecourse approach to vaccination. We aimed to draw conclusions from the Arab population to make health system recommendations that can benefit other underserved populations who suffer from low vaccine rates.

## Methods

### Study population and recruitment

We recruited mothers, teenagers and health care professionals from Northern Israel, where 52% of Arab residents of Israel live. We selected municipalities that reflected the range of religions, rural/urban differences, and geography to ensure that there would be wider representation of the different population groups within the Arab population. We decided not to include Druze participants in the study, since they are ethnically, culturally and religiously a distinct group. Even though the Druze faith developed out of Islam, Druze do not identify as Muslims and have been recognized since 1957 by the Israeli government as a distinct ethnic community at the request of its communal leaders [21].

#### Health care professionals

Health care professionals who worked directly with vaccinations among Arabs were recruited with the help of the Ministry of Health (MOH) Northern Region Office, who provided a contact list of nurses from Tipat Halav (Well-baby Family Health Clinics) and school nurses from the selected municipalities, as well as local and regional public health leads from the MOH.

#### Mothers and teenage girls

Mothers with at least one child 15 years or younger were recruited through purposeful sampling. Teenagers 13–15 years who were eligible to receive the HPV vaccine were recruited. Teens and mothers were identified with the assistance of heads of community centers and local community organizations in the selected towns, where mothers and teens were engaged in recreational and educational activities. To initiate contact, we communicated with the heads of community centers in the selected towns, who in turn contacted mothers and teenagers. Upon receiving approval to share their phone numbers with our research team, the lead researcher reached out to participants by phone. During these calls, the study was explained in detail, verbal consent was obtained, and arrangements for interview times and locations were made. Participants received an information sheet in Arabic that fully explained all aspects of participation, including the right to withdraw at any time from the research. Participants signed a written informed consent form. The consent form for the teenagers was signed by both the teenager and parent. We did not recruit fathers because mothers are typically the parent responsible for Tipat Halav visits and vaccinations.

### Topic guide development

We conducted semi-structured interviews using a topic guide that comprehensively discussed factors affecting vaccine update among Arabs in Israel. The topic guide asked about knowledge, attitudes, views and perceptions related to childhood vaccination; their experience with vaccination; health system enablers to vaccination; challenges (if any), or fears. Interview protocols were reviewed and revised accordingly for clarity, cultural appropriateness and sensitivity by a research advisory committee, comprised of the Head of Health Promotion Department in the MOH, a nurse with years of experience working with vaccination in the Arab community, and a representative from the leading civil society organization on Arab health issues. The guides were pilot tested on one health professional, one mother, and one teenager to ensure that the questions were appropriate before starting the data collection process. Both health care professionals and mothers were asked about MMR and HPV. Nurses who worked only in the Tipat Halav clinic were able to offer insights only about MMR, since they do not deal with HPV given in the schools. Nurses in the schools administer both vaccines, hence were able to relate to MMR and HPV. Teenagers were only asked about HPV.

### Data collection

All data was collected as part of an EU funded project, RIVER-EU (Reducing Inequalities in Vaccine uptake in the European region – Engaging Underserved communities). Interviews were conducted in Arabic between April 2022 through August 2022. Questions were open ended, and probes were used to elicit more information. Nurse interviews were held in clinics, and those with mothers and teenagers in community centres, participants’ homes or via Zoom according to participant preference. All but two interviews were held in person. Interviews concluded once full and complete understanding of the research topic was achieved, and data saturation had been met [22].

### Data analysis

Interviews were recorded, transcribed and translated to English. We back translated a sample of the transcripts to ensure translation accuracy. We analyzed the transcripts using thematic content analysis, closely following the six steps identified by Braun and Clarke [23]. The main researcher became familiar with the data by reading the transcriptions for each interview and making notes, recording general feedback and noting patterns. Initial codes were generated, and the sections of the transcripts were highlighted. After coding we generated themes and illustrated them with quotes from the interviews. The themes were then mapped to the WHO health system building blocks, according to six building blocks: (1) service delivery; (2) health workforce; (3) information; (4) medical products; (5) vaccines and technologies; (6) financing, and leadership/governance [24]. This framework has previously been used to investigate barriers and enablers to public health programs [7, 25, 26]. We did not identify themes that we could not map to the building blocks.

## Results

### Participant demographics

We interviewed 21 health care professionals (mean age 44 years old and 16 years of experience), 16 mothers (mean age 43 years old) and 16 teenagers (mean age 14). Table 1 describes their socio-demographic characteristics.

**Table 1:** Demographic data on health care professionals

**Health Professionals** (***n* = 21)**	**Age**	n (%)
	25–34 years	2 (10)
	35–44 years	8 (38)
	45–54 years	7 (33)
	55–64 years	4 (19)
	**Years’ Experience**	
	0–5 years	1 (5)
	6–10 years	5 (24)
	11–15 years	5 (24)
	16–20 years	4 (19)
	More than 20 years	6 (28)
	**Population work with**	
	Muslims	8 (38)
	Christians	-
	Mixed	13 (62)
	**Vaccination Administered**	
	MMR	10 (48)
	HPV	-
	Both	11 (52)
	**Place of employment**	
	Policy maker	1 (5)
	Well-baby Clinic (Tipat Halav)	10 (47)
	School	9 (43)
	Well-baby clinic and school	1 (5)
**Mothers** *N* = 16	**Age**	
	25–34 years	-
	35–44 years	10 (63)
	45–54 years	6 (37)
	55–64 years	-
	**Religion**	
	Christian	7 (44)
	Muslim	9 (56)
	**Education**	
	High school	7 (44)
	Bachelor’s degree	6 (37)
	Master’s degree or more	3 (19)
	**Number of children**	
	1	-
	2	1 (6)
	3	11 (69)
	4 or more	4 (25)
	**Employment status (yes/no)**	
	Yes	12 (75)
	No	4 (25)
Teenagers (*n* = 16)	**Age**	
	14 years	9 (56)
	15 years	7 (44)
	**Religion**	
	Christian	6 (38)
	Muslim	10 (62)
	**Received HPV Vaccine**	
	Yes	16 (100)
	No	0

### Health system enablers to uptake of childhood immunizations

A variety of enablers were identified for the high uptake of MMR and HPV among the Arab community. These are described below and summarized in Table 2.

**Table 2:** Health system enablers, according to the different target populations

Enabler	Health Professionals		Mothers		Teenagers	
**Building Block 1: Service delivery**						
Access to vaccination is universal and highly available	✓	MMR HPV	✓	MMR HPV		
Vaccinations are given in schools (MMR and HPV)	✓	MMR HPV	Mixed responses		✓	HPV
Routine vaccinations are tested and tried- generation to generation	✓	MMR HPV	✓			
Welcoming clinic environment	✓	MMR	✓	MMR		
**Building Block 2: Health work force**						
Health professionals are from same culture.	✓	MMR HPV	Mixed responses		✓	HPV
Very high trust in health system, particularly family physician and nurses	✓	MMR HPV	✓			
Belief that health professionals are most knowledgeable when it comes to vaccination.	✓	MMR HPV				
Personable characteristics and patience of nurses	✓	MMR HPV	✓			
**Building Block 3: Health information systems**						
Services are available in Arabic	✓	MMR HPV			✓	HPV
Healthy system set up- reminders- facilitate vaccination and friendly environment	✓	MMR	✓	MMR		
**Building Block 5: Financing**						
Health system provides vaccination at no extra costs	✓	MMR HPV				
**Building Block 6: Leadership and governance**						
Vaccinations given equally to minority populations	✓	MMR HPV	✓			

### Service delivery enablers

#### Access to vaccination is universal and highly available

Nurses and mothers both reported widely available vaccines and no difficulties reaching clinics. All municipalities had at least one Tipat Halav clinic and larger cities more with clinics spread across different geographic locations with no transportation barriers. Nurses emphasized that there was great ease in setting up appointments for vaccinations and they were always available at the clinic when needed, as illustrated by this quote:

We make it so easy to access our services. When a mother tells me she can’t attend at a specific time, we do everything we can to help her- even if it means coming in very early or staying later. (Tipat halav nurse, female, 47 years)

#### Vaccinations are delivered in schools

Nurses felt that vaccinating in school was a key contributor to high HPV vaccine uptake. They emphasized that when students are vaccinated with peers, it encourages uptake and minimizes hesitations and fears.

Most mothers felt schools were an appropriate and convenient setting for vaccination since the nurse and the school did all the “work.” A few mothers raised concerns related to hygiene and infection control of vaccinations delivered in the school environment. Most mothers indicated that vaccination outside of school would be more difficult and lead to delays in vaccination.

Teenagers also viewed schools as a good place for vaccinations and did not raise concerns. Seeing their peers go through the same process was reassuring and encouraged them to get vaccinated:

I like getting the vaccine in school with my friends. When I see everyone getting the shot it makes me feel more comfortable…. It’s not just something that I have to go through on my own. (teenager, 14 years)

#### Vaccines are tried and tested

Nurses and mothers recurrently reported that the long-standing availability of routine vaccines was an important enabler. Mothers reported no fears or hesitations in vaccinating their children because they were under the impression generations of women before them were vaccinated - although this is unlikely to be the case since MMR only became available in 1988 and HPV in 2007. Mothers associated vaccination with the elimination of deadly diseases:

It’s safe, and everyone has gotten it. My mother, my grandmother, myself. And it obviously has protected us from getting serious diseases. It has been there for years. When I hear of my Jewish friends and colleagues questioning vaccines- I think how can they not just give their kids the vaccine. How can they make such a decision and take the responsibility of risking their kids’ health? (mother 43 years, 3 kids)

#### Welcoming clinic environment

Most nurses and mothers reported a welcoming physical environment as an enabler. Most clinics had play areas, which helped keeping children and their siblings busy while the nurse meets with the mothers:

I usually have problems keeping my older kids occupied when I have an appointment. In Tipat Halav though, they have a play area. So I could take my older kids and know they could play safely while I focused on getting the baby vaccinated and checked by the nurse. (mother, 42 years, 4 kids)

### Health workforce enablers

#### Health professionals are from the same culture

Nurses expressed that sharing the patient’s cultural background increased feelings of safety and trust, which promoted vaccination. Mothers and teenagers also emphasized that the shared background made it easier to ask questions:

I prefer that she be Arab. It’s easier to express myself and I know the nurse understands our culture. Even though Hebrew is not a problem, it’s just easier to express yourself in your native language. (mother, 44 years, 3 kids)

#### Very high trust in health professionals

Nurses indicated their patients had high trust in their routine vaccine recommendations. Mothers trusted that if health professionals recommend HPV and MMR vaccine, it was for the child’s well-being:

I don’t know how to explain it. They just believe in it and believe in us. They trust us- whatever we tell them. They deeply believe that we act for their own good and wellbeing. (Tipat halav nurse, female, 54 years).

#### Belief that health professionals are most knowledgeable when it comes to vaccination

Mothers indicated that health professionals were the first resource they turned to when seeking vaccine information. They did not report turning to others, such as religious leaders, for advice on vaccination. Although they did search the internet and social media about general health issues, they often did not trust these resources. One mother stated:

I use social media a lot- but not for something as important as information about vaccines for my children. If I need information, I ask my nurse at Tipat Halav or my family physician. These are the people I can trust. (mother, 43 years)

Teens also highly trusted health professionals, despite indicating that they did not feel like they had a role in the actual decision-making process. The majority of teenagers thought the vaccine was mandatory and were not given the option to refuse vaccination. They indicated that the parents were the decision makers and expressed that nurses and parents knew what was best for them.

I don’t think it is up to me to decide if I want a vaccine. It is something mandatory. If the doctor and nurse says I need to get it, they know best and I have to get it. My parents will agree, and then I have to get it to protect my health. (teenager, 15 years)

Nurses also felt they were resources that Arab parents turned to when they had questions about vaccines:

Jewish parents prefer to be the decision makers. They will tell me- with all due respect to you as a nurse, but I know what is best for my child. I never see this with Arab parents. They tell me, you know what is best when it comes to vaccines for my baby. If you recommend it, I will take it. (Tipat halav nurse and school nurse, female, 37 years).

#### Nurses’ patience and kindness

Mothers and teenagers recognized that nurses’ patience and kindness promoted vaccination. They noted that nurses were always willing to answer questions patiently, with a positive attitude. One mother stated:

I remember the nurse was really kind. She wasn’t like- yalla (Arabic word for let’s go), let’s get the injection. She took her time with the kids, give them time to get accustomed to the environment, play, and chat. Then gradually she would give them the vaccine. (mother, 42 years)

Nurses also recognized the importance of being patient with parents and children.

When we are with them, we devote all our attention and time. We give each one her time, unlike at the HMO (health maintenance organization) where they go and the doctors are always in a hurry. Here we don’t rush. (Tipat halav nurse, female 50 years)

### Health information system enablers

#### Services and health information available in Arabic

While healthcare professionals recognized the importance of printed and digital materials being available in Arabic, they thought it more important that verbal instructions be provided in Arabic, since parents are more likely to listen to nurses than read materials. Mothers expressed preference of an Arab health professional but did not mention educational materials in Arabic as an enabling factor. Teenagers however, felt it was helpful:

It helps that the information they give us is in Arabic. I remember the school nurse gave us a lecture in Arabic and she handed out information in Arabic. I skimmed it- didn’t read so much- but I’m sure if it were in Hebrew I would not even have looked at it! (teenager, 14 years)

#### Reminders and recall

Nurses emphasized that automatic reminder systems helped keeping parents up-to-date regarding vaccination. Parents received automatic appointment reminders vias SMS, with a link option to change their appointment if needed. Nurses reported this system was effective and reduced workload and mothers found the system useful and user-friendly:

I get reminders about the appointments. It’s not like they don’t care if you don’t come. They remind you before the appointment…. And if for some reason you don’t go, they send you another reminder. They call as well if you don’t respond. They also call after the shot and follow-up to check to make sure the child is ok. (mother, 42 years, 3 kids)

### Financing enablers

#### Free vaccination

Nurses believed that offering vaccination for free was an important enabler, particularly because of the low socio-economic status of many Arab families. Nurses thought payments would lower vaccine uptake rates, not because the parents do not *want to vaccinate*,* but because many might not be able to*:

If mothers had to pay extra for vaccines it would definitely affect rates. When we talk about meningococcal vaccine [not included in the schedule] and tell them there is a 600 NIS payment, this is high for people…. And because they have to pay extra, the response rate is low. (Tipat halav nurse, female, 47 years)

In addition to direct costs, nurses also highlighted the importance of opening hours matched to parents’ working schedule:

Opening the clinics on Fridays (weekend day in Israel) really was a game changer. This allowed working mothers to easily come in on their day off. And when they can’t, we do everything to help find a time suitable for them…. They just contact me and pick a day and I try to be flexible. (Tipat Halav nurse, female, 33 years).

Mothers however were less clear about financial enablers, did not share similar thoughts. They indicated they would vaccinate their child regardless of costs, recognizing the importance of vaccines. This mother said:

Even if I had to pay extra for it, whatever it costs, as long as I’m convinced it would help my child. (mother, 39 years)

Mothers did not raise indirect costs such as transportation or taking time off work as an issue, but did emphasize that opening the clinic on Fridays or Sundays, their day off of work, and flexibility around working hours facilitated vaccination.

### Leadership and governance enablers

#### Vaccinations given equally to minority populations

Nurses and mothers all agreed that health services were offered equally to all residents of the State, regardless of ethnicity or religion. Even mothers who perceived societal discrimination felt health was different:

Usually we suffer from discrimination and feel as a minority group that we are treated differently by the State institutions. However, with health I don’t feel this…we get the same treatment, regardless of religion or ethnicity. At least here we are treated equally. Maybe it is because there are always Arabs wherever you go for health care. (mother, 41 years)

## Discussion

Although belonging to a minority group is generally associated with low vaccine coverage [2], coverage among Arabs in Israel is higher than the national average. Although this has been documented for several years, our study is the first to identify factors that contribute to enabling such a phenomenon. These included easy access, vaccine availability, school-based programs, friendly clinic environment, trust in health professionals and their knowledge, services and information in Arabic, shared cultural background between healthcare professionals and patients, personable characteristics of nurses, reminder systems, free vaccines, and an equitable delivery process. Our study suggests that a combination of good access to and delivery of vaccination, delivered by a culturally competent, available, accessible respectful workforce can enable disadvantaged minorities to achieve high vaccine coverage, in particular in a cultural context where the population trusts and follows medical advice and parental authority over teenagers is the norm. While we repeatedly found that sharing a cultural background with the healthcare worker vaccinating was an enabler, understanding and describing this enabling mechanisms was outside the scope of this study and would warrant further research in its own right.

At the health system level, some of the enablers are quite universal, regardless of specific vulnerable population, including service delivery enablers (vaccine access and availability, delivery of vaccination in schools [6, 27], health care workforce enablers (health care professionals from the same culture [6, 7], high trust in health care providers [28, 29]), health information system enablers (availability of culturally appropriate services [7], reminder systems [30]), financial costs [31], and feelings of equity [6, 30].

Other enablers are more specific to the Arab population and are highly affected by cultural and social norms. “Doctor knows best” is a cultural norm that has been found in other studies among Arab or Muslim populations [32]. Unlike in the Jewish Israeli population, Arab mothers have absolute trust in health care professionals and do not question their recommendations. Despite Arabs in Israel reporting lower trust in political institutions, like the Ministry of Health [33, 34], they had greater trust in health care than Jews [35, 36]. This high trust, especially in Tipat halav nurses, has been previously reported as an enabler to vaccination [34]. Reasons for such high trust in Tipat halav nurses is that the nurses treat the mothers as individuals and devote time to a variety of topics, including vaccinations [34].

Evidence from other Arab communities across the world are conflicting. Arab American women have reported mistrust towards physicians and health care professionals in the United States, particularly when language and financial concerns were a barrier [38]. It is possible that there less health care professionals with similar culture or backgrounds in the U.S., which would affect the level of trust. This might be due to the way the health system is organized in the U.S., which is very different than the Israeli health care system. On the other hand, Arab parents in the United Arab Emirates indicated almost complete trust in their child’s doctor [39].

Social and cultural norms can strongly influence health seeking behaviours, particularly vaccination decisions. Some parents rationalized their decision to vaccinate because it was the ‘norm’ in their culture [40]. In Arab society, parent’s responsibility to keep their children healthy is a social norm that influences their vaccination decisions. The Arab community is a conservative society that tends to adopt behaviors that are socially acceptable and within the norm [41]. Social pressures placed on parents to vaccinate children was mentioned as a reason for high uptake rates. No association was made between HPV and sexual activity, even among highly educated mothers. The HPV vaccine is presented as a vaccine against cancer caused by the papilloma virus, with little emphasis on the mode of transmission. This is in line with the WHO’s strategy to emphasize HPV as a cancer vaccine rather than focusing on sexual transmission [42].

Mothers and teenagers had limited knowledge of HPV, a finding in line with other studies [20]. Teenagers expressed limited roles in the HPV vaccine decision-making process. They were simply told there was a vaccine, parents signed the form, and they were vaccinated. There was no other discussion about the issue. Most teenagers believed the vaccine was mandatory. This is in line with cultural values in Arab societies, which are characterized as collective and authoritarian, in which parents make important family decisions and their consent is required [43]. In contrast, in some western societies, self-consent by minors is an ongoing debate that is gaining support as a possible means to increase uptake since requirement for written parental consent has been shown to be a barrier to HPV vaccination [44, 45]. However, among more conservative societies self-consent by minors may result in parental resistance, and conflicts between parents and adolescents [44].

Findings in the literature on the relationship between religion and vaccination are conflicting. Studies examining parent’s HPV vaccination beliefs among Christians, Jews, Hindu, and Muslims show inconsistent results regarding whether religion has an impact on the decision to receive the HPV vaccine [46, 47]. In our study, religion was not reported as a barrier. Rather, nurses and mothers agreed that religion, regardless of Islam or Christianity, was supportive of vaccination. Muslim mothers emphasized that verses in the Koran directly support vaccination to protect health. Religious leaders were not consulted about vaccinations. Nurses also working with Jewish communities indicated major differences, emphasizing that Rabbis played a key role particularly with the HPV vaccine and were routinely consulted on vaccine related issues.

In general, Arabs in Israel have a lower socio-economic status than the general Jewish population [13, 14]. While nurses indicated that providing vaccination at no extra cost was a significant enabler for the community and payments would lower uptake rates, particularly because many Arab families have multiple children, mothers did not mention costs as an enabler, possibly because it is the norm to receive childhood vaccinations at no extra costs. Regardless of what the true impact of free vaccination might be, expressed willingness to pay for vaccines illustrates how protecting children from risk was placed above financial concerns. Israel, and within it the Arab population, is a child-oriented society. Arab women in Israel are allocated the health caretaker role of family members [48]. Mothers’ needs come second to their children’s needs and being a ‘good mother’ means prioritizing their child’s health [49].

Arabs in Israel face discrimination in many areas of life. Although they have formal equality under the law, in practice they are largely an underprivileged minority with a history of disadvantage in income, education, and employment and under-represented in the governmental, judiciary, civil service, and other areas of civic life [33]. However, perceived discrimination among Arabs was not associated with health [50], possibly because Arabs are over-represented in the health care workforce. According to a 2020 MOH report Arabs who comprise about 21% of the country’s population, constitute 46% of recipients of medical licenses [51].

Throughout the years, the Ministry of Health has developed and implemented programs and policies focusing on the Arab community to create the situation of trust and adherence. The high level of trust is not a spontaneous occurrence, but rather the outcome of a sustained and prolonged process of trust building, with specific efforts that begin in the maternity wards and years of education campaigns promoting routine child vaccinations (Ministry of Health, personal communication). As indicated by mothers in this study, generational influences play a pivotal role, with mothers and grandmothers instilling confidence in vaccine safety, thereby fostering a cultural norm. Consistent with these findings is the different attitude towards COVID-19 vaccination, which was perceived as something “new” and not part of the inter-generational cultural norm of vaccination, leading to lower confidence from Arab mothers with this vaccine [37]. Here, despite having high levels of trust in the Tipat Halav nurses, the lack of experience with the vaccine, of the lack of available evidence on long-term side effects, led to greater reluctance in vaccination uptake.

Our findings have several implications for vaccination policy among disadvantaged minority populations. Recommendations for implementation of interventions to improve immunizations among disadvantaged population groups are mapped according to the WHO health system building blocks framework; these are summarized in Table 3. Our findings can also help promote other aspects of health among the Arab population in Israel. While Arabs have good health indicators in terms of child vaccination, the population sees poor health indicators in many areas, including higher mortality rates from chronic diseases and higher prevalence of type 2 diabetes [52]. Barriers related to ease and access to service, translated services and materials, health care workforce characteristics, and financial costs are not unique to vaccine services, and are observed across health services when treating underserved populations, such as diabetes or general health care [53, 54]. Minimizing these barriers at the system level should be a priority for policymakers, yielding benefits beyond a single programme. However, it is important to take into account that vaccination has become a salient and sensitive topic with very strong beliefs and attitudes at the individual level that does not exist in other fields of medicine. Although there may be valuable lessons learnt, the findings are not necessarily transferrable particularly to curative approaches and require separate studies to in fact determine specific enablers and barriers.

**Table 3:** Recommendations for strategies mapped to the WHO building blocks to promote MMR and HPV vaccination among disadvantaged, minority populations

WHO Building Block	Recommendations
Service delivery	•Provide vaccinations in the school system. •Develop strategies so that vaccinations are widely available and accessible to the community- this includes providing in locations close to the population group, and ensure that health clinics providing vaccinations offer “opening” hours that are varied (wide availability to appointments, different days, and different hours). •Provide a welcoming clinic environment, with play area for young children, including siblings of children coming for vaccination
Workforce	•Provide cultural competence training to HCPs so they can better understand the specific needs of these disadvantaged, minority populations. •Ensure the workforce is diverse and reflects the populations being served. •Train HCPs on the importance of kindness, sensitivity, and patience in delivering vaccination services.
Health information system	•Set up automatic reminder and recall systems to facilitate vaccination process where not in place. •Provide vaccination, educational materials, and health services in minority populations’ native language AND ensure materials are culturally appropriate.
Financing	•Offer free MMR and HPV vaccination.
Governance/ Leadership	•Offer vaccination services equally, without actual or perceived discrimination, to disadvantaged populations.

Triangulating viewpoints from health professionals, mothers, and teenagers was a major strength of the study that improves the completeness and reliability of the information generated. Including these three groups allowed us to understand the viewpoints in terms of enablers and barriers of those giving the vaccination (health professionals), those making the decision to vaccinate (mothers), and those receiving the vaccine (teens). We recruited participants from different religions, education levels, employment status, and diverse town population size and urban/rural town characteristics to try to get as many different perspectives as possible. While qualitative research does not purport to be representative in the statistical sense, the diversity of our participants makes the findings relevant to a large part of the Arab population of Northern Israel where the study was based.

Arabs in the South, particularly the Bedouin population, have different characteristics and living situations than those in Northern and central Israel, with significant barriers to accessing healthcare [55]. Thus, our findings are not directly applicable to the Bedouins in the South. Another limitation of our study was the lack of male representation among all participants included. With such a high uptake rate of vaccination among this community, we were not able to recruit unvaccinated participants. While our study took place during the COVID 19 pandemic, the data collection took place outside of the initial period that saw disruption in the routine vaccine programme.

Each population’s needs and unique characteristics should be taken into account when developing interventions and must be considered. Health professionals and policymakers need to consider the specific cultural context of the target populations they are working when implementing vaccine programs. Nevertheless, evidence from the literature and findings among other minority populations we studied as part of the RIVER-EU project (https://river-eu.org/), a consortium project aimed at improving vaccine uptake among minority groups, which this study belongs to, revealed that minority populations share many similar health system barriers and enablers to health care in terms of vaccination access, including ensuring educational materials and health services are provided in minority population’s native language and in a culturally appropriate manner, by health professionals who have received adequate cultural competence training, ensuring that guidelines and clear and health care service is available and accessible, and offered at no cost (or highly subsidized) to the community [56]. Likewise, Israeli society is comprised of many different groups with different cultural backgrounds, speaking many different languages. Some of the enablers we found, including providing widely available and accessible services, in the same language as the target population, by staff aware of cultural sensitivity specific to the population, delivered by caring and compassionate health care workers who are trained in communication, and at no cost are universal. Policy makers need to learn the importance of knowledge transfer to improve services across different populations and ensuring that health services are delivered in a way that is tailored to the needs of specific population groups.

## Conclusions

Using the WHO health system building blocks framework, we identified health system enablers to MMR and HPV vaccination among Arabs in Israel, a minority that achieves high vaccine coverage despite socio economic deprivation. Our study suggests that the combination of access, delivery workforce and governance, in the right cultural context enables minority populations, who traditionally achieve lower vaccine coverage than the general population, to attain high vaccine coverage. While the cultural context is a key factor in creating positive deviance, several recommendations, predominantly those not rooted deeply in culture, may be transferred to other disadvantaged minority groups when developing vaccination policies and program. These include providing vaccination and health services in populations’ native language and by a health professional from the same culture, or alternatively a workforce trained for culture competence; develop strategies such that vaccines are widely available and accessible to the community; set up automatic reminder systems to facilitate the vaccination process; offer vaccinations in the school system; train professionals about the importance of patience and kindness; set up clinics that provide a friendly, safe, warm environment; provide vaccinations at no cost; and finally framing routine child vaccinations as the norm. These findings may help inform interventions aimed at improving vaccine coverage among underserved communities in other settings.

## Data Availability

Transcripts from the interviews are confidential and not publicly available. A subset of deidentified data are available after review of the request by a RIVER-EU committee chaired by the coordinator (UMCG).

## Abbreviations

HPV: Human Papillomavirus
MMR: Measles, Mumps, and Rubella (MMR)
WHO: World Health Organizations
MOH: Ministry of Health
